# Association between the ZJU index and risk of new-onset non-alcoholic fatty liver disease in non-obese participants: a Chinese longitudinal prospective cohort study

**DOI:** 10.3389/fendo.2024.1340644

**Published:** 2024-02-09

**Authors:** Keyang Zheng, Yuzhe Yin, Hang Guo, Linlin Ma, Rufei Liu, Tianzhu Zhao, Yuxuan Wei, Zixu Zhao, Wenli Cheng

**Affiliations:** ^1^ Center of Hypertension, Beijing Anzhen Hospital, Capital Medical University, Beijing, China; ^2^ Sixth Clinical Medical School, Capital Medical University, Beijing, China; ^3^ Department of Education, Beijing Anzhen Hospital, Capital Medical University, Beijing, China; ^4^ Department of Cardiovascular Medicine, Beijing Anzhen Hospital, Capital Medical University, Beijing, China

**Keywords:** non-alcoholic fatty liver disease, the ZJU index, fatty liver index, non-obese population, obesity

## Abstract

**Background:**

Non-alcoholic fatty liver disease (NAFLD) is increasingly observed in non-obese individuals. The ZJU (Zhejiang University) index has been established as a new and efficient tool for detecting NAFLD, but the relationship between the ZJU index and NAFLD within non-obese individuals still remains unclear.

**Methods:**

A *post-hoc* evaluation was undertaken using data from a health assessment database by the Wenzhou Medical Center. The participants were divided into four groups based on the quartile of the ZJU Index. Cox proportional hazards regression, Kaplan-Meier analysis and tests for linear trends were used to evaluate the relationship between the ZJU index and NAFLD incidence. Subgroup analysis was conducted to test the consistency of the correlation between ZJU and NAFLD in subsgroups. Receiver operative characteristic (ROC) curve analysis was performed to evaluate the predictive performance of the ZJU index, compared with the Atherogenic index of plasma (AIP) and Remnant lipoprotein cholesterol (RLP-C) index.

**Results:**

A total of 12,127 were included in this study, and 2,147 participants (17.7%) developed NAFLD in 5 years follow-up. Participants in higher ZJU quartiles tended to be female and have higher liver enzymes (including ALP, GGT, ALT, AST), GLU, TC, TG, LDL and higher NAFLD risk. Hazard Ratios (HR) and 95% confidence intervals (CI) for new-onset NAFLD in Q2, Q3, and Q4 were 3.67(2.43 to 5.55), 9.82(6.67 to 14.45), and 21.67(14.82 to 31.69) respectively in the fully adjusted model 3. With increased ZJU index, the cumulative new-onset NAFLD gradually increased. Significant linear associations were observed between the ZJU index and new-onset NAFLD (p for trend all<0.001). In the subgroup analysis, we noted a significant interaction in sex, with HRs of 3.27 (2.81, 3.80) in female and 2.41 (2.21, 2.63) in male (P for interaction<0.01). The ZJU index outperformed other indices with an area under the curve (AUC) of 0.823, followed by AIP (AUC=0.747) and RLP-C (AUC=0.668).

**Conclusion:**

The ZJU index emerges as a promising tool for predicting NAFLD risk in non-obese individuals, outperforming other existing parameters including AIP and RLP-C. This could potentially aid in early detection and intervention in this specific demographic.

## 1 Background

Non-alcoholic fatty liver disease (NAFLD) is a spectrum of liver disorders characterized by excessive hepatic fat accumulation in the absence of excessive alcohol consumption or any other secondary causes diagnosed by liver biopsy (1). Considering the global obesity and metabolic syndrome epidemics, NAFLD is becoming the leading cause of chronic liver damage and cannot be understated due to its rising prevalence, accounts for 25% worldwide (2) and 15%-40% in Asia (3), epidemiologically. The disease continuum of NAFLD spans from simple steatosis or fatty liver, progressing to non-alcoholic steatohepatitis (NASH) accompanied by varying degrees of fibrosis, and can eventually culminate in cirrhosis (4). Identifying individuals at risk early in the disease trajectory is crucial for timely intervention and prevention of its progression.

Although it’s widely known that NAFLD is commonly associated with obesity, it is reported that 40% of the NAFLD patients worldwide (5) and 17.5% of those in Chinese population are non-obese (6). As they often overlook the need for clinical evaluation for NAFLD and are likely to be overlooked in risk assessments for the condition, this population may pose greater challenges than obese individuals. Existing studies have proposed limited indices, including AIP (Atherogenic index of plasma) and RLP-C (Remnant lipoprotein cholesterol), as potential predictors for risk assessment in non-obese population (7, 8). First proposed by Wang et al. in a cross-sectional study involving 9,602 Chinese participants, the Zhejiang University (ZJU) Index proved to be a proficient tool for screening NAFLD (AUC=0.822) (9, 10). Subsequent researches have confirmed the association between the ZJU index and the incidence of NAFLD in both Chinese and international populations, demonstrating superior predictive accuracy compared to the aforementioned indices (11, 12). However, its efficacy in non-obese populations remains invalidated.

Therefore, this study aims to assess the relationship between the ZJU index and the incidence of NAFLD in non-obese populations and explore whether this relationship exhibits sex differences. Additionally, we aim to compare the effectiveness of the ZJU index with some preexisting indicators (AIP, RLP-C) in predicting the onset of NAFLD.

## 2 Methods

### 2.1 Study design and population

We derived data from the Dryad data repository at http://datadryad.org/ under the doi: 10.5061/dryad.1n6c4.14. Spearheaded by the Wenzhou Medical Center of Wenzhou People’s Hospital, this longitudinal study focused on non-obese participants screened from individuals who underwent health examinations in this hospital from January 2010 to December 2014. The findings revealed a direct link between levels of lipoprotein cholesterol (LDL-C) and an elevated likelihood of NAFLD (diagnosed by ultrasound and accompanied with alcohol consumption ≤140g/week for men and ≤70g/week for women) (13). And this study has been approved by the Ethics Committee of Wenzhou People’s Hospital.

Our goal was to examine the association between the ZJU index and the new-onset NAFLD outcomes in non-obese individuals. All participants in the database who completed the 5-year follow-up were included. Exclusions were made based on following criteria: (1) participants diagnosed with NAFLD at baseline; (2) BMI >25kg/m^2^; (3) alcohol consumption >140 g/week for men and >70 g/week for women; (4) on antihypertensive, antidiabetic, lipid-lowering medications or any other known causes of chronic liver disease; (5) LDL-C >3.12mmol/L; (6) incomplete data which is unable to calculate the ZJU index.

### 2.2 Ultrasonographic diagnosis of NAFLD

Certified technicians employed abdominal ultrasonography to diagnose NAFLD, adhering to the diagnostic guidelines set by the Chinese Liver Disease Association (13). NAFLD was characterized by a subdued far-field echo and a pronounced enhancement of the near-field echo in the liver area, more intense than in the kidney or spleen areas.

Positive indicators for NAFLD included any of the following: indistinct representation of the intrahepatic ductal structure, slight to noticeable enlargement of the liver with softened and rounded edges, diminished blood flow signals maintaining typical distribution, and a vague or incomplete visualization of the outer layer of the right liver lobe and diaphragm.

### 2.3 Demographic and laboratory evaluation

Medical history and alcohol use were documented by trained physicians. BMI (kg/m2), which is used to evaluate body fat content, was derived from the individual’s weight in kilograms divided by their square of height in m^2^. Blood pressure was assessed with an automated sphygmomanometer in a quiet setting and seated position. The suite of biochemical tests included measurements for albumin (ALB), alanine aminotransferase (ALT), aspartate aminotransferase (AST), fasting plasma glucose (FPG), blood urea nitrogen (BUN), creatinine (Cr), uric acid (UA), total cholesterol (TC), TG, HDL-c, and LDL-c. A sophisticated automated system (Abbott AxSYM) was employed to conduct these tests using conventional methodologies. The ZJU index is calculated as BMI + FBG + TG + 3x ALT/AST (+2 if female) (9). RLP-C was calculated as TC–HDL-C+LDL-C (8). And the AIP was calculated by the logarithm of TG/HDL-C mole ratio base 10 (7).

### 2.4 Follow-up and outcome evaluations

New-onset NAFLD was the outcome of this analysis. Hepatic ultrasonic examinations were conducted in a blinded manner (as at baseline) to assess whether the outcome target has been achieved. Annual reviews were conducted throughout the observation period, utilizing the same evaluation procedures as the initial assessment.

### 2.5 Statistical analysis

The participants were divided into four groups according to the ZJU index quartiles. Continuous variables were expressed as mean (standard deviation) or median (Q1-Q3) based on the distribution of data. In the test for differences, data with a normal distribution were analyzed using ANOVA analysis; for data with a skewed distribution, the Kruskal-Wallis H test was used. All categorical data were expressed as frequency (percentile). Chi-square test or Fisher test were applied to compare the categorical variables. Cox proportional hazards regression was applied to evaluate Hazard Ratios (HRs) and their 95% Confidence Intervals (CIs) to discern the association between the ZJU index quartiles and the occurrence of the new-onset NAFLD in three models. Model 1 was unadjusted, model 2 accounted for age. To select adjustment variables for further modeling, covariates were selectively introduced into the basic model or removed from the complete model to assess the impact on the regression coefficients of the ZJU quartiles, with those altering the coefficients by more than 10% identified as adjustment variables. Model 3 was further adjusted for parameters including age, ALP, GGT, TC, ALB, DBIL, CR and BUN. Tests for linear trends were conducted using quartiles of ZJU index values as continuous variables. The Kaplan-Meier analysis was used to display the cumulative incidence of NAFLD in different ZJU groups. Subgroup analysis was conducted to estimate the consistency of the effect in different groups including sex, age (<60, ≥60 years), BMI (<21.8, ≥21.8 kg/m²), SBP (<140, ≥140 mmHg), and ALT (<16, ≥16 U/L). The BMI subgroup was divided using the median value, while other variables were categorized based on commonly used clinical thresholds. Receiver operative characteristic (ROC) analysis curve was performed to evaluate the predictive performance of the ZJU index, comparing with AIP and RLP-C. The area under the curve (AUC) of each method was calculated. All statistical processes were conducted using the R software package (V.4.0; The R Foundation; http://www.R-project.org). A two-tailed p-value of less than 0.05 was considered statistically significant.

## 3 Results

### 3.1 Baseline characteristics of the participants

**Figure 1:**
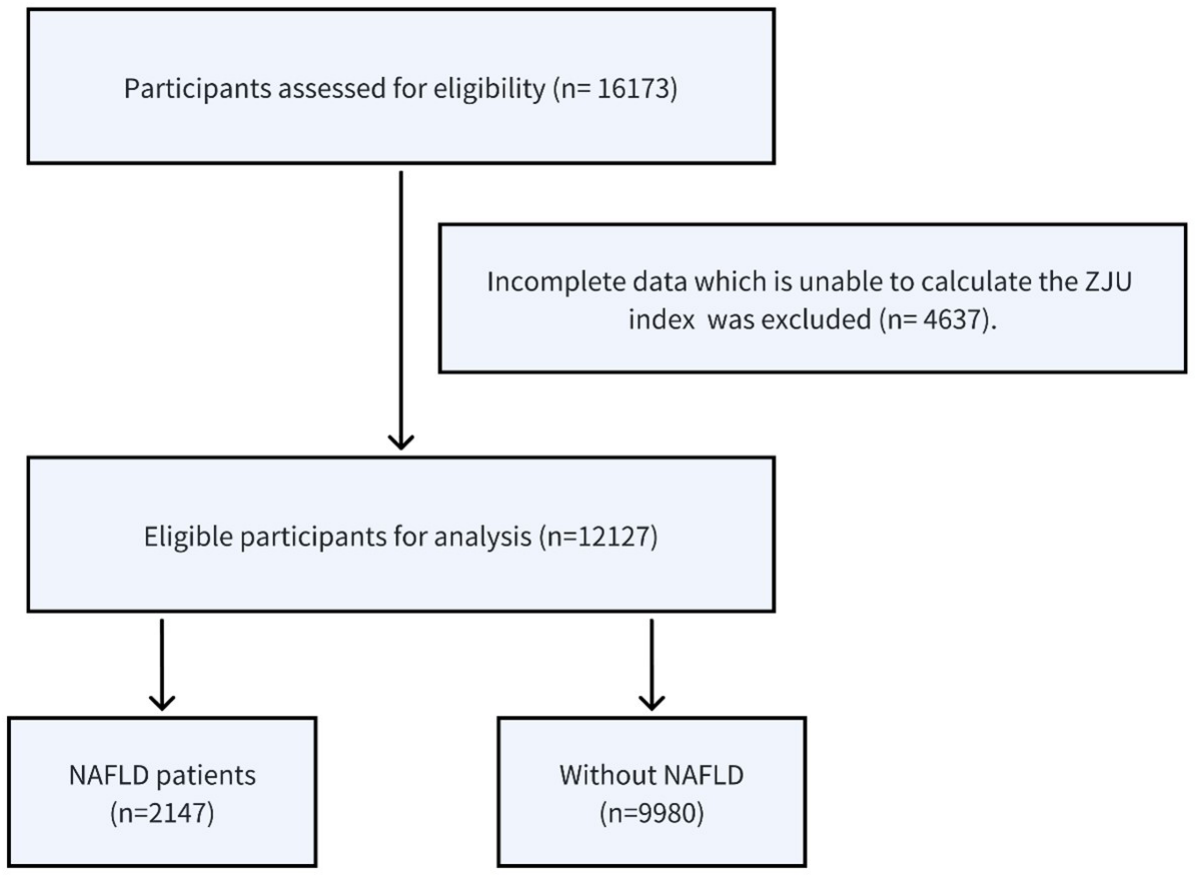
Inclusion and exclusion flow chart for the longitudinal cohort.

A total of 16,173 non-obese participants were initially enrolled in the study, and among these, 12,127 individuals met the eligibility criteria and were included in the analysis (**Figure 1**). There were 6646(54.8%) male and 5481(45.2%) female, and the average age of the participants was 43.29 ± 14.96. All participants completed the 5-year follow-up examination and 2,147(17.7%) participants developed NAFLD.

The baseline characteristics of the participants included according to the ZJU index quartiles were shown in **Table 1**. Participants in the higher ZJU index group were more likely to be female and have higher BMI, SBP, DBP, liver enzymes (including ALP, GGT, ALT, AST), BUN, Cr, UA, GLU, TC, TG, LDL and higher NAFLD risk, but lower levels of DBIL and HDL-C than those in the lower ZJU index groups (all P<0.001). There was no statistically significant difference in other indicators among different groups (all p value>0.05).

**Table 1:** Baseline characteristics of the participants in different ZJU groups.

Variables	ZJU index groups				p Value
	Q1	Q2	Q3	Q4	
n	3032	3031	3032	3032	
Male, n (%)	2148 (70.8)	1709 (56.4)	1632 (53.8)	1157 (38.2)	<0.001
ZJU index, mean (SD)	27.60 (1.35)	30.50 (0.63)	32.63 (0.63)	35.75 (1.98)	<0.001
Age, mean (SD)	43.19 (15.54)	43.26 (15.12)	43.72 (14.69)	42.97 (14.41)	0.249
BMI (Kg/m2), mean (SD)	19.16 (1.34)	21.16 (1.16)	22.56 (1.20)	23.57 (1.06)	<0.001
SBP (mmHg), mean (SD)	114.90 (15.58)	120.67 (15.83)	125.09 (16.61)	128.96 (16.40)	<0.001
DBP (mmHg), mean (SD)	69.56 (9.35)	72.78 (9.91)	75.09 (10.11)	77.79 (10.38)	<0.001
ALP (U/L), median [IQR]	64.00 [53.00, 77.00]	67.00 [56.00, 81.00]	71.00 [59.00, 85.00]	76.00 [63.00, 90.00]	<0.001
GGT (U/L), median [IQR]	17.00 [14.00, 22.00]	20.00 [16.00, 26.00]	23.00 [18.00, 32.00]	31.00 [22.00, 49.00]	<0.001
ALT (U/L), median [IQR]	12.00 [10.00, 16.00]	15.00 [12.00, 19.00]	18.00 [14.00, 23.00]	24.00 [17.00, 33.00]	<0.001
AST (U/L), median [IQR]	20.00 [17.00, 23.00]	21.00 [18.00, 24.00]	22.00 [19.00, 26.00]	23.00 [20.00, 28.00]	<0.001
ALB (g/L), mean (SD)	44.55 (2.80)	44.45 (2.83)	44.49 (2.67)	44.67 (2.77)	0.016
TB (µmol/L), mean (SD)	12.19 (5.07)	12.21 (5.35)	12.18 (4.88)	12.34 (4.92)	0.702
DBIL (µmol/L), median [IQR]	2.00 [1.50, 2.70]	2.00 [1.40, 2.70]	2.00 [1.40, 2.70]	1.80 [1.30, 2.50]	<0.001
BUN (mmol/L), median [IQR]	4.20 [3.40, 5.10]	4.40 [3.63, 5.30]	4.50 [3.77, 5.41]	4.50 [3.80, 5.40]	<0.001
Cr (µmol/L), median [IQR]	74.00 [64.00, 87.00]	80.00 [68.00, 93.00]	85.00 [72.00, 96.00]	88.00 [77.00, 97.00]	<0.001
UA (µmol/L), mean (SD)	254.78 (82.80)	281.81 (85.24)	306.00 (85.86)	331.05 (85.82)	<0.001
GLU (mmol/L), mean (SD)	4.93 (0.43)	5.08 (0.47)	5.20 (0.59)	5.64 (1.32)	<0.001
TC (mmol/L), mean (SD)	4.43 (0.71)	4.58 (0.71)	4.64 (0.70)	4.79 (0.80)	<0.001
TG (mmol/L), median [IQR]	0.88 [0.71, 1.11]	1.04 [0.81, 1.35]	1.23 [0.95, 1.66]	1.69 [1.22, 2.43]	<0.001
HDL-c (mmol/L), mean (SD)	1.60 (0.34)	1.51 (0.35)	1.41 (0.34)	1.27 (0.32)	<0.001
LDL-c (mmol/L), mean (SD)	2.12 (0.46)	2.26 (0.46)	2.33 (0.46)	2.38 (0.46)	<0.001
NAFLD, n (%)	48 (1.6)	197 (6.5)	573 (18.9)	1329 (43.8)	<0.001

BMI, body mass index; SBP, systolic blood pressure; DBP, diastolic blood pressure; ALP, alkaline phosphatase; GGT, gamma glutamyl transferase; ALT, alanine aminotransferase; AST, aspartate aminotransferase; ALB, albumin; TB, total bilirubin; DBIL, direct bilirubin; BUN, blood urea nitrogen; CR, serum creatinine; UA, uric acid; GLU, glucose; TC, total cholesterol; TG, total triglycerides; HDL-c, high-density lipoprotein cholesterol; LDL-c, low-density lipoprotein cholesterol; NAFLD, non-alcoholic fatty liver disease.

### 3.2 Relationship between ZJU index and new-onset NAFLD in different sex

To further investigate the relationship between ZJU index group and the new-onset NAFLD, Cox’s proportional hazards regression analyses were conducted (**Table 2**). In unadjusted model 1, the HR for NAFLD was 3.80 (95% CI 2.77 to 5.21), 10.94 (95% CI 8.15 to 14.69) and 27.88 (95% CI 20.91 to 37.19) for Q2, Q3 and Q4, respectively. After adjusting for age, the HR was similar in model 1. In the fully adjusted model 3, we adjusted for parameters including age, ALP, GGT, TC, ALB, DBIL, CR and BUN, and the HRs of the ZJU index and NAFLD in Q2, Q3 and Q4 were 3.67 (95% CI 2.43 to 5.55), 9.82 (95% CI 6.67 to 14.45) and 21.67 (95% CI 14.82 to 31.69).

**Table 2:** Association between ZJU index groups and incidence of NAFLD.

ZJU groups	Model 1	Model 2	Model 3
	HR (95%CI) P value		
Male			
Q1	1	1	1
Q2	4.10 (2.93, 5.75) P<0.01	4.11 (2.93, 5.76) P<0.01	3.70 (2.37, 5.77) P<0.01
Q3	12.38 (9.04, 16.94) P<0.01	12.37 (9.04, 16.93) P<0.01	10.29 (6.80, 15.56) P<0.01
Q4	27.71 (20.33, 37.78) P<0.01	27.52 (10.19, 37.51) P<0.01	19.58 (12.96, 29.57) P<0.01
P for trend	2.72 (2.55, 2.90) P<0.01	2.71 (2.54, 2.89) P<0.01	2.41 (2.21, 2.63) P<0.01
Female			
Q1	1	1	1
Q2	5.25 (2.08, 13.28) P<0.01	5.25 (2.08, 13.26) P<0.01	5.98 (1.81, 19.73) P<0.01
Q3	16.02 (6.57, 39.07) P<0.01	16.00 (6.56, 39.01) P<0.01	16.40 (5.19, 51.84) P<0.01
Q4	64.52 (26.77, 155.47) P<0.01	64.42 (26.73, 155.25) P<0.01	55.15 (17.67, 172.12) P<0.01
P for trend	3.78 (3.36, 4.24) P<0.01	3.77 (3.36, 4.23) P<0.01	3.27 (2.81, 3.80) P<0.01
Total			
Q1	1	1	1
Q2	3.80 (2.77, 5.21) P<0.01	3.81 (2.78, 5.22) P<0.01	3.67 (2.43, 5.55) P<0.01
Q3	10.94 (8.15, 14.69) P<0.01	10.96 (8.16, 14.71) P<0.01	9.82 (6.67, 14.45) P<0.01
Q4	27.88 (20.91, 37.19) P<0.01	27.97 (20.97, 37.31) P<0.01	21.67 (14.82, 31.69) P<0.01
P for trend	2.78 (2.63, 2.93) P<0.01	2.78 (2.63, 2.93) P<0.01	2.50 (2.33, 2.69) P<0.01

Model 1 was not adjusted.

Model 2 was adjusted for age.

Model 3 was adjusted for age, ALP, GGT, TC, ALB, DBIL, CR, BUN.

ALP, alkaline phosphatase; GGT, gamma glutamyl transferase; TC, total cholesterol; ALB, albumin; DBIL, direct bilirubin; CR, serum creatinine; BUN, blood urea nitrogen.

We further analyzed the association between each ZJU index group and NAFLD according to sex. We found that regardless of sex, the ZJU index was strongly associated with new-onset NAFLD. When ZJU group was set as an continues variable, the HR (95%CI) in Model 3 was 2.41 (2.21, 2.63) with a P-value of <0.01 for males and 3.27 (2.81, 3.80) with a P-value of <0.01 for females.

Significant linear associations were observed between the ZJU index and new-onset NAFLD in both sexes and the entire cohort (p for trend all<0.001).

### 3.3 Kaplan-Meier curves analysis

**Figure 2:**
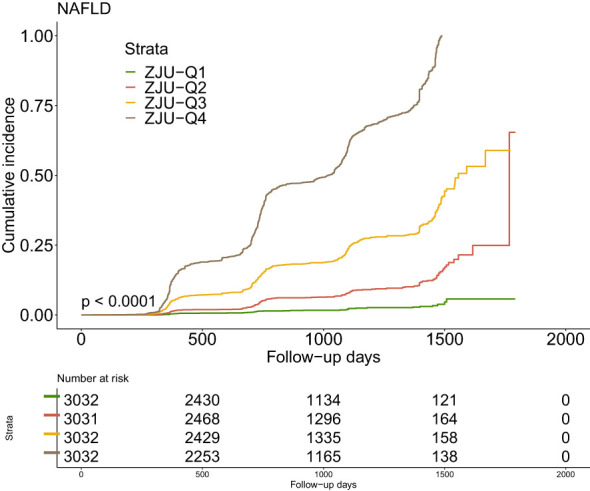
Kaplan–Meier estimation of new-onset NAFLD by ZJU quartiles. NAFLD, non-alcoholic fatty liver disease.

The Kaplan-Meier curves were shown in **Figure 2** illustrating the accumulative incidence of new-onset NAFLD stratified by ZJU index quartiles. There was a distinct disparity in NAFLD risk across various the ZJU index levels (P<0.001). A higher ZJU index was associated with a progressive increase in the incidence of new-onset NAFLD, with the highest quartile undoubtedly exhibiting the maximum risk.

### 3.4 Subgroup analysis for the risk of new-onset NAFLD by ZJU index

To test the consistency in different groups, we analyzed the relationship between the ZJU index (per one increase) and new-onset NAFLD through a subgroup analysis, as outlined in **Table 3**. The chosen subgroups were based on sex, age (<60, ³60 years), BMI (<21.8, ³21.8 kg/m²), SBP (<140, ³140 mmHg), and ALT (<16, ³16 U/L). A significant interaction was observed between sex and new NAFLD diagnoses (p for interaction<0.01). In females, the ZJU index showcased a higher predictive power. The respective HRs in females and males were 3.27 (2.81, 3.80) and 2.41 (2.21, 2.63). For other subgroups, no significant interactions with the ZJU index were significant in predicting NAFLD, with all interaction p > 0.05.

**Table 3:** Subgroup analysis of the impact of ZJU index on NAFLD incidence.

Variables	ZJU groups (per group)		
	HR, 95%CI	P value	P for interaction
Sex			<0.01
Female	3.27 (2.81, 3.80)	<0.01	
Male	2.41 (2.21, 2.63)	<0.01	
Age			0.27
<60	2.57 (2.36, 2.79)	<0.01	
>=60	2.32 (2.00, 2.67)	<0.01	
BMI			
<21.8	2.30 (1.99, 2.65)	<0.01	0.84
>=21.8	2.34 (2.09, 2.61)	<0.01	
SBP			0.12
<140	2.58 (2.37, 2.80)	<0.01	
>=140	2.19 (1.87, 2.56)	<0.01	
ALT			0.05
<16	2.45 (2.14, 2.80)	<0.01	
>=16	2.22 (2.03, 2.43)	<0.01	

Model was adjusted for age, ALP, GGT, TC, ALB, DBIL, CR, BUN.

BMI, body mass index; SBP, systolic blood pressure; ALT, alanine aminotransferase.

To further explore whether the sex differences in the relationship between the ZJU index and incident NAFLD varied across different age groups, we conducted an analysis stratifying by age with a cutoff at 50 (**Table 4**). Notably, the findings revealed a marked interaction between sex and the incidence of new NAFLD cases in the cohort aged above 50 years, with the interaction p-value <0.01.

**Table 4:** Subgroup analysis for sex in different age groups of ZJU index on NAFLD incidence.

Variables	ZJU groups (per group)		
	HR, 95%CI	P value	P for interaction
Age<50			0.28
Female	2.61 (2.01, 3.39)	<0.01	
Male	2.24 (1.98, 2.53)	<0.01	
Age>=50			<0.01
Female	3.50 (2.93, 4.17)	<0.01	
Male	2.60 (2.30, 2.94)	<0.01	

### 3.5 Predicted performance of the ZJU index by ROC

**Figure 3:**
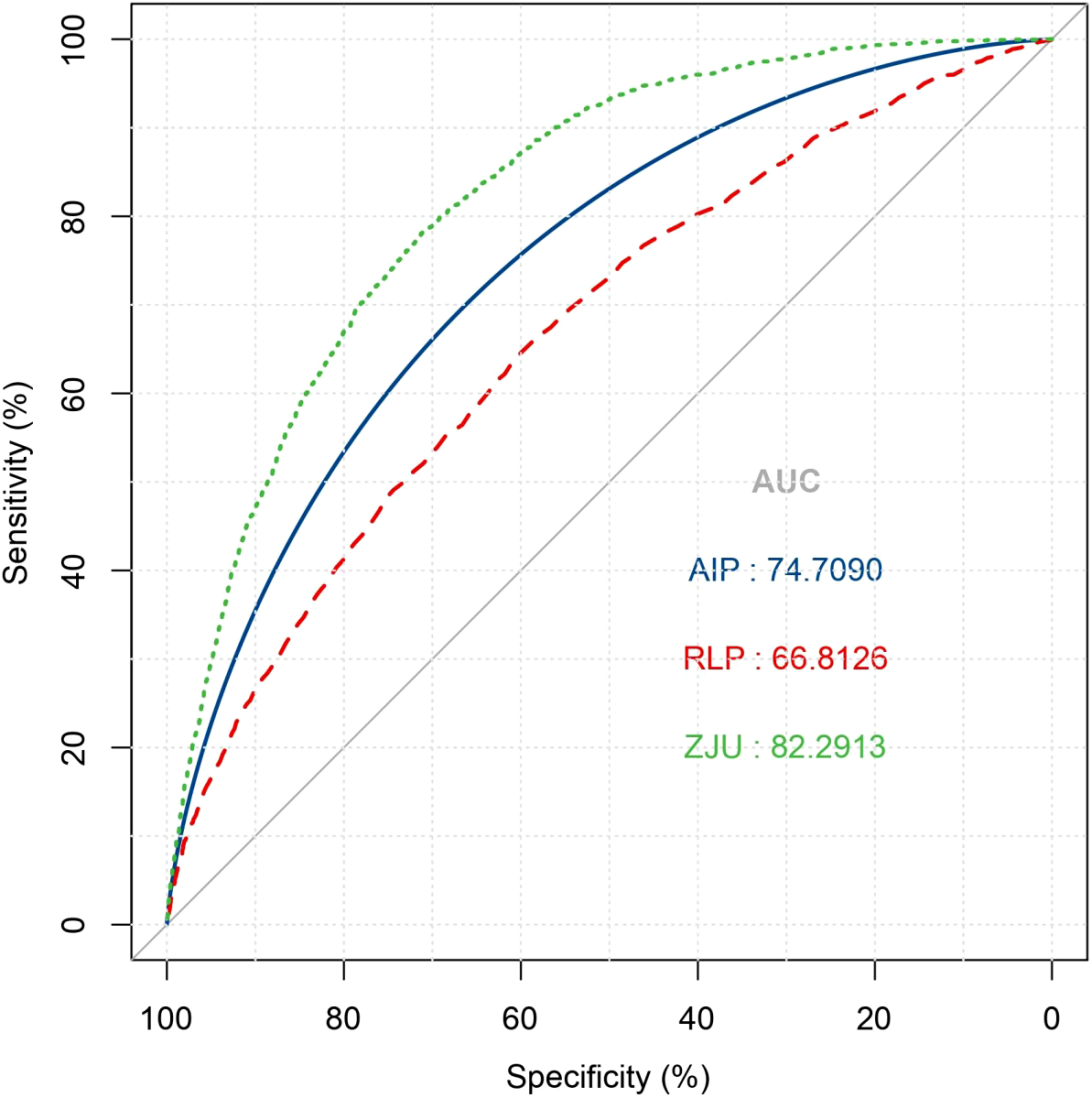
Receiver operative characteristic curve of ZJU index, AIP and RLP-C for identifying NAFLD. NAFLD, non-alcoholic fatty liver disease; AIP, Atherogenic index of plasma; RLP-C, Remnant lipoprotein cholesterol.

In the receiver operating characteristic analysis using NAFLD indices, including AIP and RLP-C, to identify NAFLD, the ZJU index exhibited the highest area under the curve (AUC = 0.823) followed by AIP (AUC = 0.747) and RLP (AUC = 0.668), respectively. (**Figure 3**).

## 4 Discussion

In this longitudinal study, we observed that the ZJU index is a significant risk factor for new-onset NAFLD in non-obese populations, showing superior predictive efficacy compared to existing indices AIP and RLP-C. Notably, there was a significant interaction between sex and new NAFLD diagnoses, with the correlation being more substantial in the female subgroup. In clinical practice, the ZJU index presents a valuable tool for assessing NAFLD risk in non-obese populations, aiding in the early identification of individuals at high risk for NAFLD and facilitating early interventions to improve their prognosis.

The ZJU index, initially proposed by Wang et al. in a cross-sectional study involving 9,602 Chinese participants who underwent hepatic ultrasound examination to diagnose NAFLD, is recognized as a proficient tool for screening NAFLD in the Chinese population (AUC=0.822) (9). Several prior studies have validated that the ZJU index effectively predicted NAFLD in both domestic and international populations. In a cross-sectional study involving 19,804 Chinese adults, the ZJU index, fatty liver index, hepatic steatosis index, lipid accumulation product, and visceral adiposity index of each participant were calculated. The ZJU index showcased superior diagnostic accuracy in identifying NAFLD with an AUC of 0.925 (95% CI 0.919-0.931), outperforming four other indexes. This superior diagnostic performance was consistent in age and sex subgroups, showing the ZJU index as a reliable and efficient tool for NAFLD detection (11). In another cohort involving 28,729 Chinese adults without fatty liver disease at baseline, the ZJU index was found to be significantly associated with an elevated risk of developing fatty liver disease over a median follow-up of 3.01 years. As the ZJU quartiles increased, the HRs increased correspondingly, with 4.87 (4.24-5.59) for women and 6.23 (5.56-6.98) for men (12). Moreover, validation in North American settings was evident in a study involving 107 obese women, which revealed an AUC of 0.742 for the ZJU index, emphasizing its applicability in predicting NAFLD among this population (10). However, few studies have examined its correlation with NAFLD in non-obese individuals. Our findings on the ZJU index align with these past studies, underscoring its reliable predictive value for new-onset NAFLD in non-obese group. Notably, while previous studies predominantly focused on obese or overweight participants, our research breaks new ground by delving into the relationship between the ZJU index and NAFLD in non-obese individuals.

Furthermore, when we conducted subgroup analyses on various factors, we noted a significant interaction in the sex subgroup with respect to the ZJU index. There was a larger effect size of the association between the ZJU index and new-onset NAFLD risk in women than men. The result may be attributed to the distinct metabolic profiles between males and females. Women generally exhibit a gynoid gluteo-femoral subcutaneous fat distribution, while men tend to have an android visceral fat distribution (14). On the other hand, estrogen, prevalent in females, provides protection against NAFLD (15). In this process, estrogen-related receptor alpha (ERRα) is a critical modulator in the downstream of estrogen/ERα signaling, particularly influencing hepatic very low-density lipoprotein (VLDL) assembly and secretion, with its dysfunction leading to hepatosteatosis and potential progression to NAFLD, especially in men and postmenopausal women (16). In additional analyses, we employed the age of 50 years as a surrogate marker for the onset of menopause. he results demonstrated a significant interaction between sex and the association between the ZJU index for new-onset NAFLD in the population aged over 50. Conversely, this interaction was not significant in the population under 50 years. In the research by Li et al. (12), the findings revealed a higher HR for men, at 6.23 (5.56-6.98), compared to women, who had an HR of 4.87 (4.24-5.59). This conclusion stands in contrast to the results of our study. However, in further subgroup analyses, both the ≤50 and >50 age groups exhibited higher HRs for women compared to men. Notably, in the >50 age group, the gender difference in HRs was more pronounced, aligning with the conclusions of our study, which suggested that the ZJU index may be a more effective predictor of NAFLD in postmenopausal women.

Previous research indicated that the development of fatty liver is associated with disruptions in lipid metabolism, anomalies in glucose processing, and impaired functionality of adipose tissues (17, 18). The components of ZJU index, including ALT, AST, TG, and FPG, serve as representative markers for hepatic metabolic irregularities (19, 20). Besides, in a study of 3,329 Chinese adults, the ZJU index revealed a profound link with insulin resistance (IR). With ascending ZJU quartiles, significant increases were observed in parameters like BP, waist circumference, and cholesterol levels, while a decline was noted in HDL-C levels. The ZJU index stood out not only for signifying an amplified risk of IR in its highest quartile but also for its superior predictive accuracy, boasting ROC curve values of 0.833 in males and 0.788 in females (21). Insulin resistance acts as a risk factor for NAFLD (22). It paves the way for NAFLD both directly, by amplifying *de novo* lipogenesis, and indirectly, by boosting the free fatty acid flux to the liver due to diminished lipolysis inhibition (23). Consequently, the ZJU index emerges as an optimized predictive model that encapsulates various forecasting factors.

NAFLD is progressively identified in non-obese individuals, and these cases can sometimes have more adverse outcomes than their obese counterparts, including a quicker progression to cirrhosis (24, 25). The progression of fibrosis occurs more rapidly in lean individuals with NAFLD compared to those who have a higher BMI (24). Genetic variations in Patatin-like phospholipase domain-containing protein 3 (PNPLA3), transmembrane 6 superfamily 2 (TM6SF2) or other genes associated with steatosis could possibly explain the association between lean NAFLD and the increased risk of future development of severe liver disease (26). Moreover, early detection and intervention can be more challenging in these non-obese patients than in those who are obese. Miao et al. (8) using the same source data as ours investigated the relationship between Remnant lipoprotein cholesterol (RLP-C) and NAFLD, revealing that higher RLP-C levels were associated with an increased risk of NAFLD, with HRs for 1.6 and 1.3 in high and middle RLP-C groups. Li et al. (7) involved 16,173 participants and use the atherogenic index of plasma (AIP) to predict NAFLD. Over a span of 5 years, 14.4% of the non-obese individuals developed NAFLD. With increasing AIP values, the risk of new-onset NAFLD rose significantly, especially evident in those within the lower BMI range. In this study, when comparing the ROC curves of three indicators, the AUC values highlighted the ZJU index’s superior performance over RLP-C and AIP in predicting NAFLD. This underscores the potential of the ZJU index as a more effective tool for detecting NAFLD risk among the non-obese population.

### 4.1 Limitations

Primarily, the population studied pertains only to non-obese NAFLD individuals in China, without representation from other ethnicities or regions. Secondly, the dataset lacked variables like smoking, alcohol consumption, waist and hip circumference, which could have potentially influenced our conclusions. Current studies on the relationship between the ZJU index and NAFLD remain sparse, especially regarding non-obese populations. Hence, more extensive, multicentric research is necessary to affirm the reliability and accuracy of the ZJU index in forecasting NAFLD.

## Data availability statement

Publicly available datasets were analyzed in this study. This data can be found here: the Dryad data repository at http://datadryad.org/ under the doi: 10.5061/dryad.1n6c4.14.

## Ethics statement

The studies involving humans were approved by the ethics committee of Beijing Anzhen Hospital. The studies were conducted in accordance with the local legislation and institutional requirements. Written informed consent for participation was not required from the participants or the participants’ legal guardians/next of kin in accordance with the national legislation and institutional requirements.

## Author contributions

KZ: Methodology, Writing – original draft. YY: Methodology, Writing – original draft. HG: Formal analysis, Writing – original draft. LM: Validation, Writing – original draft. RL: Data curation, Writing – review & editing. TZ: Data curation, Writing – review & editing. YW: Visualization, Writing – review & editing. ZZ: Visualization, Writing – review & editing. WC: Project administration, Writing – review & editing.
